# Transformer-Based Tool for Automated Fact-Checking of Online Health Information: Development Study

**DOI:** 10.2196/56831

**Published:** 2025-02-21

**Authors:** Azadeh Bayani, Alexandre Ayotte, Jean Noel Nikiema

**Affiliations:** 1 Laboratoire Transformation Numérique en Santé, LabTNS Montreal, QC Canada; 2 Centre de recherche en santé publique Université de Montréal et CIUSSS du Centre-Sud-de-l’Île-de-Montréal Montreal, QC Canada; 3 Department of Management, Evaluation and Health Policy School of Public Health Université de Montréal Montreal, QC Canada

**Keywords:** fact-checking automation, transformers, infodemic, credible health information, machine learning, automated, online health information, misinformation, natural language processing, epidemiology, health domain

## Abstract

**Background:**

Many people seek health-related information online. The significance of reliable information became particularly evident due to the potential dangers of misinformation. Therefore, discerning true and reliable information from false information has become increasingly challenging.

**Objective:**

This study aimed to present a pilot study in which we introduced a novel approach to automate the fact-checking process, leveraging PubMed resources as a source of truth using natural language processing transformer models to enhance the process.

**Methods:**

A total of 538 health-related web pages, covering 7 different disease subjects, were manually selected by Factually Health Company. The process included the following steps: (1) using transformer models of bidirectional encoder representations from transformers (BERT), BioBERT, and SciBERT, and traditional models of random forests and support vector machines, to classify the contents of web pages into 3 thematic categories (semiology, epidemiology, and management), (2) for each category in the web pages, a PubMed query was automatically produced using a combination of the “WellcomeBertMesh” and “KeyBERT” models, (3) top 20 related literatures were automatically extracted from PubMed, and finally, (4) the similarity checking techniques of cosine similarity and Jaccard distance were applied to compare the content of extracted literature and web pages.

**Results:**

The BERT model for the categorization of web page contents had good performance, with *F*_1_-scores and recall of 93% and 94% for semiology and epidemiology, respectively, and 96% for both the recall and *F*_1_-score for management. For each of the 3 categories in a web page, 1 PubMed query was generated and with each query, the 20 most related, open access articles within the category of systematic reviews and meta-analyses were extracted. Less than 10% of the extracted literature was irrelevant; those were deleted. For each web page, an average of 23% of the sentences were found to be very similar to the literature. Moreover, during the evaluation, it was found that cosine similarity outperformed the Jaccard distance measure when comparing the similarity between sentences from web pages and academic papers vectorized by BERT. However, there was a significant issue with false positives in the retrieved sentences when compared with accurate similarities, as some sentences had a similarity score exceeding 80%, but they could not be considered similar sentences.

**Conclusions:**

In this pilot study, we have proposed an approach to automate the fact-checking of health-related online information. Incorporating content from PubMed or other scientific article databases as trustworthy resources can automate the discovery of similarly credible information in the health domain.

## Introduction

With rapid progressions in the digital age, and the vast dissemination of textual information available online, the likelihood of coming across misinformation has surged [1,2]. Misinformation refers to information that is untrue, incorrect, or deceptive in nature [3]. It is prevalent across various domains, with social media being a particularly prominent source [4]. Indeed, many people seek health-related topics on modern platforms and websites available online [5]. Inaccurate health-related information, however, poses an even greater risk, as it can directly impact lives [6,7]. Health misinformation is considered “a health-related claim or information which is not correct due to a lack of scientific evidence or knowledge” [4,8]. The importance of trustworthy online health information became particularly clear during the COVID-19 pandemic, which triggered a new crisis known as the COVID-19 infodemic. An infodemic refers to the excessive spread of false or misleading information across both digital and physical spaces [9] causing confusion and detrimental outcomes, as it underscores the potential risks posed by inaccurate or deceptive information to individuals [3,10]. The infodemic often manifests across 4 key areas: scientific research, policy and health care practice, news outlets, and social media platforms [11]. As a result, distinguishing between true and reliable information and falsehoods has become increasingly challenging. The labor-intensive process of manually verifying information specifically in health-related fields demands expert oversight and consumes significant time [4,9,12]. Therefore, it is crucial to establish an automated fact-checking process to help users identify the accuracy of health-related information available online.

The fact-checking process involves evaluating the truthfulness of information and consists of 3 key tasks: claim detection, evidence retrieval, and claim verification [12]. The first 2 tasks can be considered as factual verification, while the third focuses on assessing the accuracy of claims, which involves distinguishing reliable information from falsehoods to establish their factual validity [13].

Several studies have explored automating the fact-checking process, primarily focusing on misinformation in the form of fake news on websites [4,14,15] or social media [2,7,16-18]. These studies have generated synthetic datasets as the gold standard to facilitate the automation of evidence-based fact-checking. Thus, they compiled datasets comprising information or claims along with their corresponding evidence from trusted sources. Models were then trained using these datasets to automate the fact-checking process [7,10,15,17-20]. To create a database of verified claims, they used methods such as modifying phrases from Wikipedia [20], manual selection of quotation sentences and handpicking of claims from health news sites [14,15,21], and automatic selection of verified claims that were manually done by experts of journalists from fact-checking websites [10]. For example, the FEVER dataset, generated by modifying sentences taken from Wikipedia, consisted of 185,400 claims [22]. PUBHEALTH is another dataset containing false, true, unproven, and a mixture of health-related claims. The dataset also had a column containing journalist-crafted, gold-standard explanations designed to substantiate the fact-check labels assigned to each claim [6,18]. While synthetic datasets provide valuable contributions to advancing automatic fact-checking efforts, they cannot fully address real-world challenges, particularly the need for real-time, dynamic information [23]. Therefore, there is a need that claims and their associated evidence to be automatically extracted [24]. A study [25] developed a Large Language Model called TrumorGPT, which addresses limitations in fact-checking by incorporating retrieval-augmented generation and using continually updated knowledge graphs. This approach uses few-shot learning, knowledge graph construction, and semantic reasoning, which enhances the model’s ability to handle fact-checking tasks effectively. Another recent survey [12] explored automated techniques for predicting the veracity of claims, relying on natural language processing, knowledge representation, and databases. This study identified common challenges in fact-checking research and emphasized the importance of information retrieval and knowledge representation, particularly due to the rapid emergence of new claims.

Therefore, a key element of fact-checking involves identifying credible sources, and for health information, leveraging up-to-date scientific literature is essential as it is widely regarded as 1 of the most trustworthy references [26]. Indeed, numerous platforms and databases provide access to health-related and scientific literature, including Google Scholar, PubMed, ScienceDirect, and Web of Science, among others. These databases can be used as a reliable source for the automation of all the processes.

Numerous organizations have established guidelines to aid users in identifying trustworthy claims [27,28] where time-consuming manual recognition plays an important role in the process. In this pilot study, we proposed a novel automated evidence-based fact-checking approach that aims to identify and confirm accurate, truthful information using scientific literature and research databases as sources of truth. This exploratory evaluation highlights how using this approach may help users measure the extent of confidence in a web page and make informed decisions about accepting the health-related information of a website. Thus, the objective was to assess the truthfulness of health-related information through an evidence-based approach, without creating a synthetic database of claims-evidence but leveraging PubMed as a reliable source of fine-grained and up-to-date health-related information.

## Methods

Approximately 1000 web pages were provided by Factually Health company on January 31, 2023. This company specializes in identifying reliable health-content websites [29]. The web pages were selected through random sampling within various disease categories to ensure a balanced dataset while minimizing the risk of overrepresentation of any single category. This approach accounted for variations in the number of available websites across disease categories. The web pages then underwent manual cleaning. Redundant pages were removed, and those unsuitable for research were excluded based on the following criteria: pages primarily featuring video content, pages related to clinical studies, pages resembling anecdotes rather than factual health information, or pages that restricted data extraction by Python (Python Software Foundation) libraries.

After this process, a dataset comprising 538 web pages was finalized. These web pages represented a diverse range of diseases, including arthritis (81 pages), chronic obstructive pulmonary disease (79 pages), COVID-19 (66 pages), hypertension (66 pages), lung cancer (70 pages), prostate cancer (66 pages), and diabetes (110 pages).

The selection of diverse disease categories was intended to minimize potential bias in the analysis. However, our previous study demonstrated that the selected diseases did not significantly impact classification results [29]. Using the URLs of each web page, the content was extracted as text files using the “justext” library in Python, to remove additional links and extraneous content from websites, such as navigation links, headers, and footers.

The process included the following three steps: (1) Classification of web page content into 3 thematic categories, semiology, epidemiology, and management by evaluating various transformer models, including bidirectional encoder representations from transformers (BERT), SciBERT, and BioBERT, as well as traditional models such as random forest (RF) and support vector machine (SVM), (2) automating the creation of PubMed queries combining “WellcomeBertMesh” and “KeyBERT” models, (3) automatic extraction of top 20 related literatures from PubMed, and (4) applying similarity checking techniques of cosine similarity and Jaccard distance to compare the content of extracted literature and web pages vectorized using BERT tokenizer. As a reliable source of truth, PubMed was a suitable choice to find evidence for health-related claims. PubMed, an open-source platform dedicated to facilitating searches and retrieval of health-related literature, encompasses over 36 million papers [30].

### Classification of Web Page Contents

One of the necessary stages before determining the veracity of a claim or information is to detect the sentences that need to be verified [31]. These claims are crucial to the content’s main point but require verification through an annotation schema and developing a benchmark for automated claim detection [14,31]. To detect sentences that need to be verified, two major steps were taken: (1) the identification of 3 thematic categories of content and (2) the classification of web page content according to these categories.

#### The Content Categories

To compare web page content with materials from the scientific literature database, it was essential to categorize the content, ensuring that comparisons were made within the relevant subject. Three distinct thematic categories have been identified for analysis: epidemiology, semiology, and management. In the epidemiology category, we included all sentences related to the statistics of a disease, the population, the frequencies, the causes, the risk assessment of the disease, and all public health-related information about the disease (eg, as of 2014, the global prevalence rate of rheumatoid arthritis was about 0.24%). In the semiology category, we considered all sentences related to signs (eg, high blood pressure is another sign of the disease) and symptoms (eg, this disease has symptoms such as pain, discomfort, weakness, fatigue). Finally, for the management category, we considered all the sentences linked to therapeutic approach (eg, drug treatment and surgical intervention, prevention, and the element of paraclinical diagnosis of diseases (eg, a complete medical examination carried out by a doctor can better determine if a person has chronic obstructive pulmonary disease and the degree of severity of the disease).

#### Manual Annotation and Model Development

Two authors (AB and AA) independently annotated 200 web pages on a sentence-by-sentence basis considering the 3 categories of epidemiology, semiology, management, and neutral until reaching a roughly balanced amount of data across all classes [32]. We used the Cohen κ score to assess the agreement between the 2 reviewers AB and AA). Any discrepancies were resolved by the third author (JNN).

Neutral sentences were those that did not correspond to any of the defined thematic categories. Table 1 shows the distribution of sentences for each category. The portable serverless text annotation tool of MedTator-1.3-11 [33] was used for the annotation process. A total of 3 transformer models of BERT, SciBERT, and BioBERT were used to classify the sentences into the 4 mentioned categories. The BERT model has demonstrated superior performance in several text classification tasks [29,34,35]. SciBERT is an extension of BERT and is trained on a vast corpus of scientific literature spanning multiple domains [36] and BioBERT is pretrained using an extensive corpus comprising PubMed abstracts (PubMed) and full-text articles from PubMed Central [37]. We have also conducted a performance comparison between the transformer models and 2 traditional machine learning models: RF and SVM.

**Table 1:** The distribution of classes.

Category	Number of sentences
Neutral	3162
Semiology	851
Epidemiology	1171
Management	1066

The “BertTokenizer” library has been used to tokenize the incoming sentences, with the following parameters: We applied a maximum sequence length of 128 to standardize the size of each input sentence. To optimize the model's hyperparameters, we applied the Bayesian optimization approach using the ‘BayesianOptimization’ library in Python. The hyperparameter tuning spaces are detailed in Table 2.

**Table 2:** Hyper-parameter tuning search space.

Hyper-parameters	Range	Best trial
Learning rate	10^–7^, 10^–2^	3×10^–5^
Weight decay	10^–5^, 10^–1^	10^-3^
Number of epochs	(1:5)	3
Batch size	(8,16,32,64)	32

### Automating PubMed Query Generation

#### Overview

Literature extraction involved identifying scientific articles within PubMed to support the process. To achieve this, the approach requires the formulation of a query by combining keywords and Medical Subject Headings (MeSH) terms, which can be extracted from web page content. This process included three steps: (1) Automating PubMed subquery creation from MeSH terms and creating a subquery using the “WellcomeBertMesh” model, (2) Automating PubMed subquery creation from keywords using KeyBert model and creating a subquery, and (3) Construction of the final query by combining the different subqueries.

#### Automating PubMed Subquery Creation Using MeSH Terms Extracted by Transformers

All the MeSH terms were extracted from the text using a pretrained model of “WellcomeBertMesh,” which takes its inspiration from “BertMesh,” which undergoes the pretraining using the entire text of biomedical publications and is built upon the foundation of the BioBert pretrained model [38]. Given that our evidence for the websites primarily comprised health-related articles from PubMed, we selected this model. Its architecture is rooted in the latest advancements in the biomedical field, prominently featuring Microsoft’s cutting-edge “PubMedBert” as its core framework [38].

**Figure 1:**
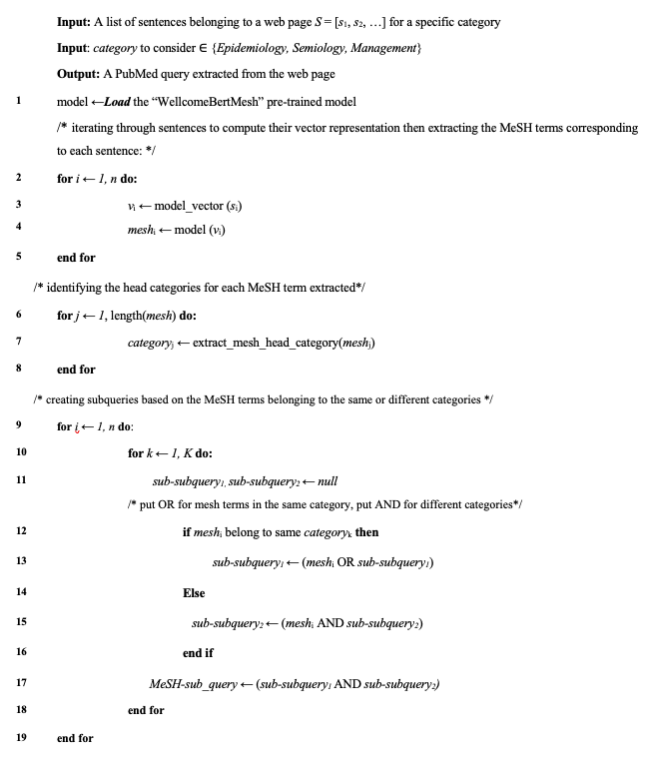
MeSH (medical subject heading) subquery builder.

To enhance the accuracy of the subquery, the identified MeSH terms were initially organized according to their MeSH categories to construct subsubqueries. The MeSH has a tree structure that is organized hierarchically, visually presenting descriptors in broader and narrower relationships. The top tier of the MeSH tree structure encompasses 19 comprehensive categories. While these terms are not included in MeSH data maintenance and distribution, they can be used to search PubMed by using the search term “category” [39]. Therefore, we have considered the MeSH terms under each head category together using the “OR” operator in this subsubquery. Then, we constructed the subquery using the “AND” operator between extracted MeSH terms in different categories. The pseudo-code for this step is presented in Figure 1.

#### Automating PubMed Subquery Creation Using Key Phrases Extracted by Transformers

The key phrases from web page contents have been extracted using the transformer model “KeyBERT” library, which is described in previous literature as having the best performance in extracting the key phrases [40], especially for long texts [41], which aligns with our need of extracting the key phrases of the scientific papers. The extracted keywords were combined with the “AND” operator to create a subquery.

**Figure 2:**
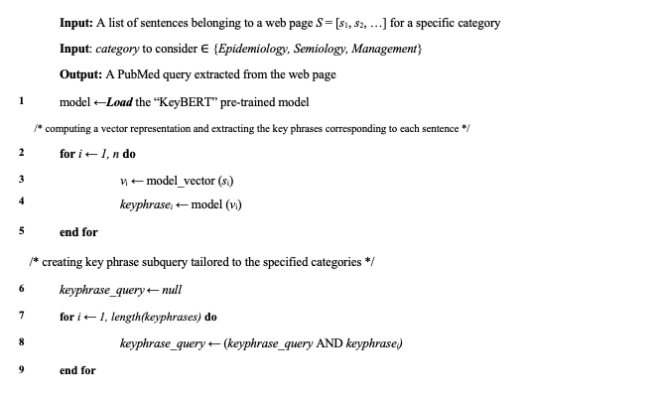
Key phrase extractor and subquery builder.

Figure 2 shows the proposed pseudo-code to extract the keywords for the creation of the subquery.

#### Construction of the Final Query

**Figure 3:**
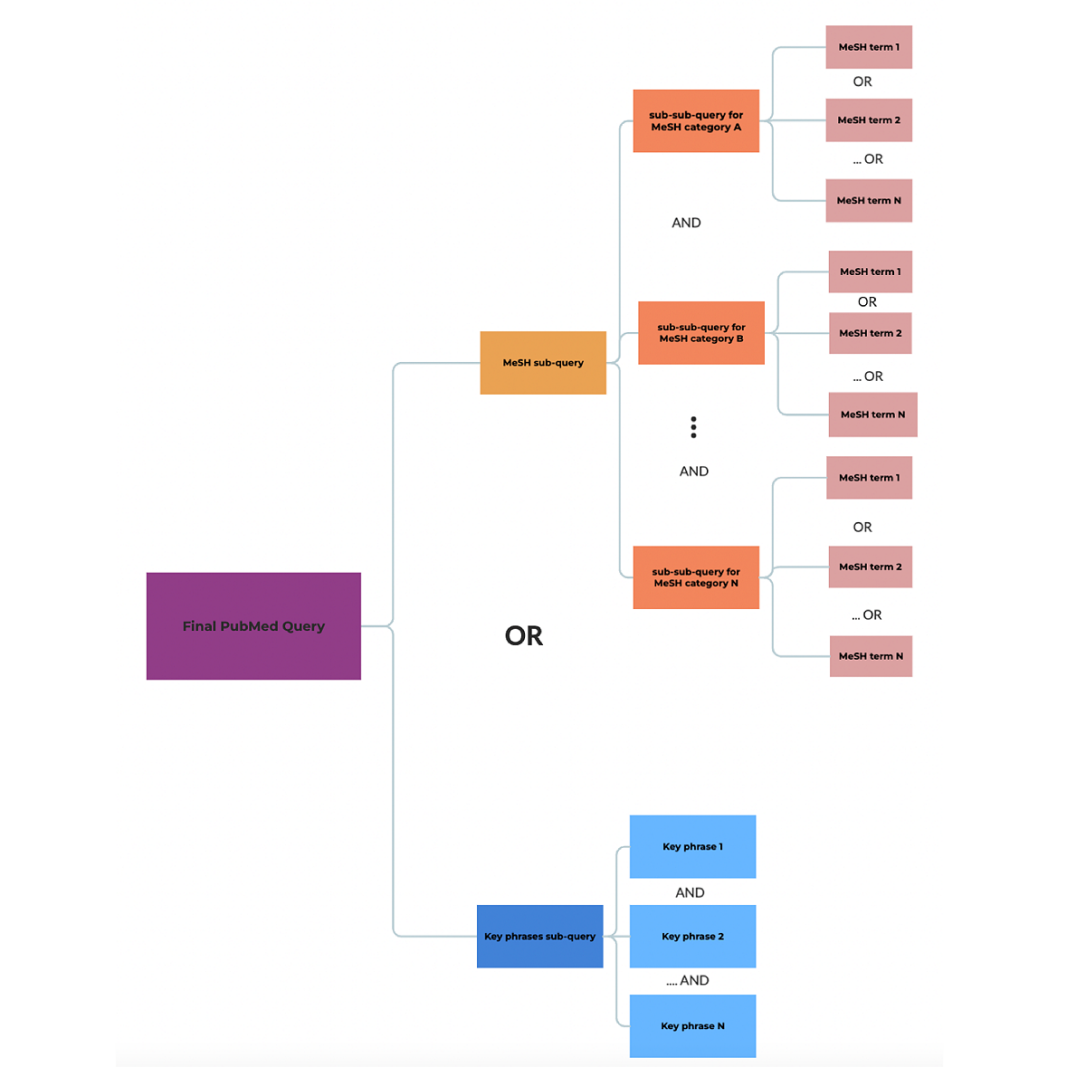
Detailed process diagram for the development of the comprehensive final PubMed query.

The subqueries extracted from the preceding processes were combined using the “OR” operator to construct the final query. Figure 3 presents a comprehensive overview of the process used to construct the final PubMed query, summarizing the structure and strategy behind its creation.

### Automating Related Literature Extraction

The final query was used to retrieve a compilation of articles, from which the top open access 20 resulting papers were extracted. The “PMC_ids” of papers were extracted using the “Entrez” library of Python that provides integrated access to PubMed Medline [42]. To evaluate the quality of our query results, we conducted a comprehensive review of the obtained full-text papers. In our assessment of the extracted papers in PubMed, those subjected to filtering within the systematic reviews and meta-analysis category exhibited more related papers to the subject of the research, compared with papers that were not subject to such filtering. Consequently, we selected them to encompass a wider range of relevant articles.

Finally, the automatically extracted papers were manually checked to be pertinent considering the title of the papers, the irrelevant papers were removed and excluded from the final process.

### Similarity Detection and Fact-Checking

For the process of computing the similarity measure between different sentences, for each disease, we randomly selected 5 web pages in our dataset. For each of the 3 predefined thematic categories in a web page, 1 PubMed query was generated and with each query, the 20 most related, open access articles within the type of systematic reviews and meta-analysis were extracted. The following steps were then carried out: (1) Categorizing the extracted related literature content based on the 3 thematic categories. This was necessary to analyze sentences (from websites and scientific articles) that are relevant to the same topics. (2) Comparing by thematic category, the content from scientific articles and web pages to identify similar sentences.

Finally, after conducting a manual evaluation of the identified similar sentences, we calculated the average number of categorized sentences for each randomly selected web page, as well as the average number of credible sentences detected. Credible sentences refer to those in the related literature that demonstrated similarity with the sentences from the web pages.

#### Categorizing the Extracted Literature

The more performant fine-tuned model on the web page contents was used to categorize literature contents into 3 thematic categories. This approach enabled us to facilitate a direct comparison between sentences sharing the same thematic context.

#### Comparing the Content From Literature and Web Pages to Identify Similar Sentences

For the sentence comparison, we used the BERT vectorizer to transform the texts into vectors. This allowed us to encode the semantic significance of sentences as numerical values, facilitating the application of different similarity detection algorithms [43].

Both scientific articles and web page sentences were transformed into vector representations, taking into account their respective thematic categories. Subsequently, each web page sentence was compared with scientific article sentences of the same category using the cosine similarity and Jaccard technique. A similarity threshold of 87% was chosen to determine sentence selection, ensuring that sentences with over 87% similarity were chosen.

**Figure 4:**
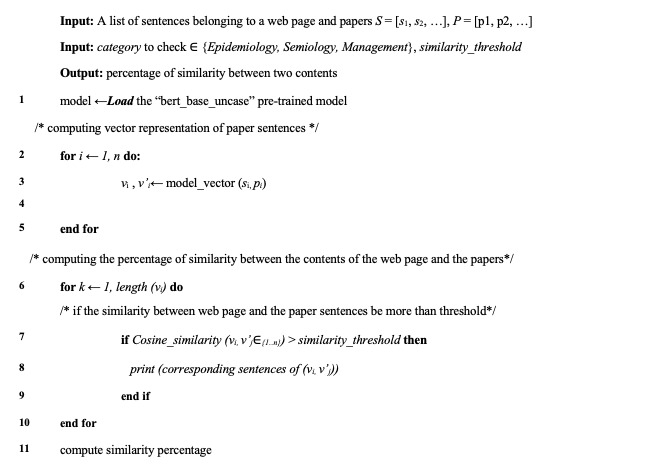
Paper similarity detection.

Figure 4 shows the proposed pseudo-code for the similarity-checking part.

For each disease, we randomly selected 5 web pages and extracted both their related papers and similar sentences. It was due to the inherent variability and specificity of medical information related to each disease. Diseases often exhibit unique characteristics, nuances, and clinical considerations. By prioritizing diseases, we aimed to provide a more granular and clinically relevant assessment of the similarity between the sentences. The outcomes, comprising sentences from the web pages and their corresponding similar sentences, underwent a manual verification by the authors to ensure semantic similarity between them. Subsequently, the proportion of semantically similar sentences between a web page and its related reference papers was calculated.

### Ethical Considerations

This research relied solely on publicly accessible data and did not involve any human or animal participants, making it exempt from the need for ethical approval. The study strictly adheres to established data privacy norms to prevent any compromise of confidentiality or privacy. In addition, the project does not include any direct involvement or interactions with individuals, thereby minimizing potential ethical issues. The University of Montreal’s Research Committee has carefully examined our methodology and affirmed that this study falls outside the scope of Medical Research Involving.

## Results

This section elaborates on the results of each part of the proposed pseudo-codes.

### Classification of Web Page Contents

The annotation process for web page contents achieved a Cohen κ score of 87% among the 2 annotators (AA and AB), indicating high agreement between the annotators and ensuring the reliability of the data used for model evaluation.

The performance of transformer-based models (BERT, BioBERT, and SciBERT) was compared to traditional machine learning models (RF and SVM) for categorizing web page content into four categories. BERT emerged as the most effective model, consistently achieving superior precision, recall, and *F*_1_-scores across all categories. Traditional models, in contrast, demonstrated lower performance, particularly in terms of *F*_1_-scores, indicating limitations in balancing precision and recall effectively.

Table 3 illustrates the performance of the classification models used to classify the content of web pages. The performance matrix includes metrics such as precision, recall, and *F*_1_-score.

**Table 3:** Performance evaluation of the BERT (Bidirectional Encoder Representations from Transformers) and machine learning models for web page content classification across considered categories.

	BERT^a^			BioBERT				SciBERT				RF^b^				SVM^c^		
Classes	Precision	Recall	*F*_1_-score	Precision	Recall	*F*_1_-score	Precision		Recall	*F*_1_-score	Precision		Recall	*F*_1_-score	Precision		Recall	*F*_1_-score
Neutral	0.96	0.93	0.95	0.88	0.83	0.85	0.85		0.81	0.83	0.51		0.92	0.66	0.72		0.81	0.77
Semiology	0.91	0.94	0.93	0.81	0.81	0.81	0.77		0.79	0.78	0.96		0.05	0.09	0.71		0.59	0.64
Epidemiology	0.92	0.94	0.93	0.80	0.76	0.76	0.75		0.74	0.75	0.8		0.1	0.1	0.69		0.62	0.65
Management	0.95	0.96	0.96	0.83	0.89	0.89	0.83		0.87	0.85	0.59		0.58	0.59	0.74		0.73	0.74

^a^BERT: Bidirectional Encoder Representations from Transformers.

^b^RF: random forests.

^c^SVM: support vector machines.

According to Table 3, among the transformer models, the BERT model had a promising performance with more than 93% recall for neutral sentences, 94% for semiology and epidemiology, and 96% for the management category. The model had an *F*_1_-score of 95% for neutral sentences, 93% for semiology and epidemiology, and 96% for management. The model had 96% precision for neutral sentences, 91% for semiology, 92% for epidemiology, and 95% for management. Also, traditional models did not have high performance, the precision values for both RF and SVM were relatively low in some classes, indicating a high rate of false positives. Also, the *F*_1_-scores for both RF and SVM were generally lower compared with the BERT model, indicating that they may not achieve a good balance between precision and recall. Therefore, the BERT model was selected for the classification of the web page contents.

**Figure 5:**
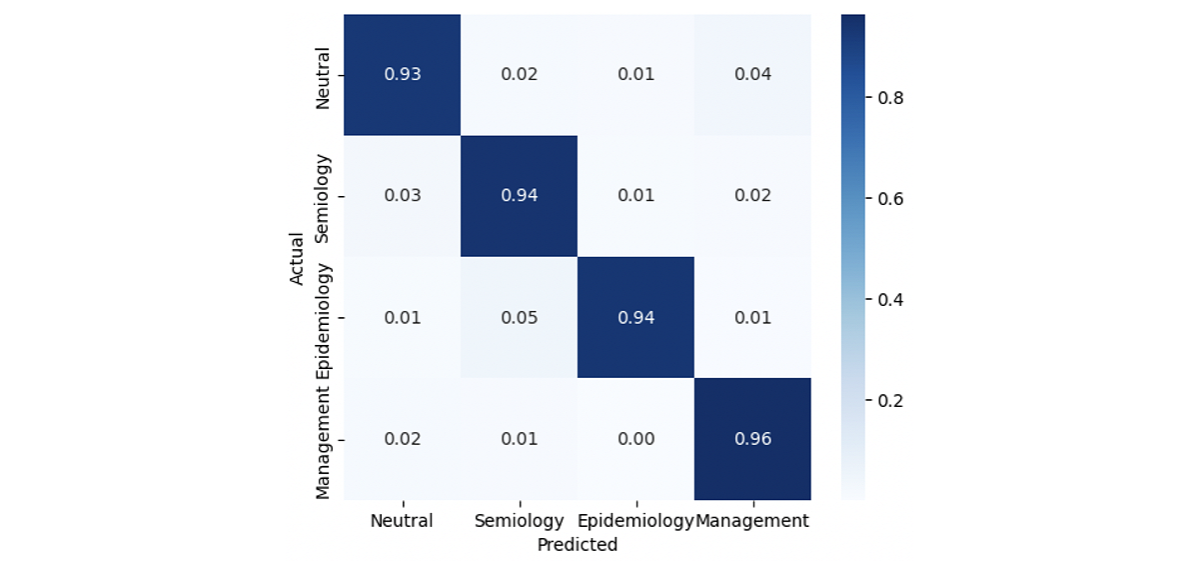
Bidirectional encoder representations from transformers model performance: confusion matrix for the classification of web page sentences into 3 thematic categories.

The confusion matrix for the BERT model is shown in Figure 5, providing a detailed visualization of its classification performance across the different categories.

Figure 5 shows the confusion matrix for the BERT classifier, which correctly classified 0.93 of the neutral sentences, 0.94 for both the semiology and epidemiology sentences, and 0.96 for management sentences as true positives.

### Automating PubMed Query Generation

To extract relevant literature for the web pages categorized thematically, a PubMed query was generated for each of the 7 diseases. Each query retrieved the 20 most related papers. The titles of the retrieved papers were manually evaluated, and less than 10% were deemed irrelevant, demonstrating the effectiveness of the generated queries. These irrelevant articles were excluded from further analysis.

This result highlights the utility of using MeSH terms and key phrases in constructing PubMed queries, which efficiently yielded pertinent literature. The generated weblinks for accessing the papers followed the format: “https://pubmed.ncbi.nlm.nih.gov/PMID/,” with PMIDs obtained directly from the PubMed queries.

### Similarity Detection and Fact-Checking

**Figure 6:**
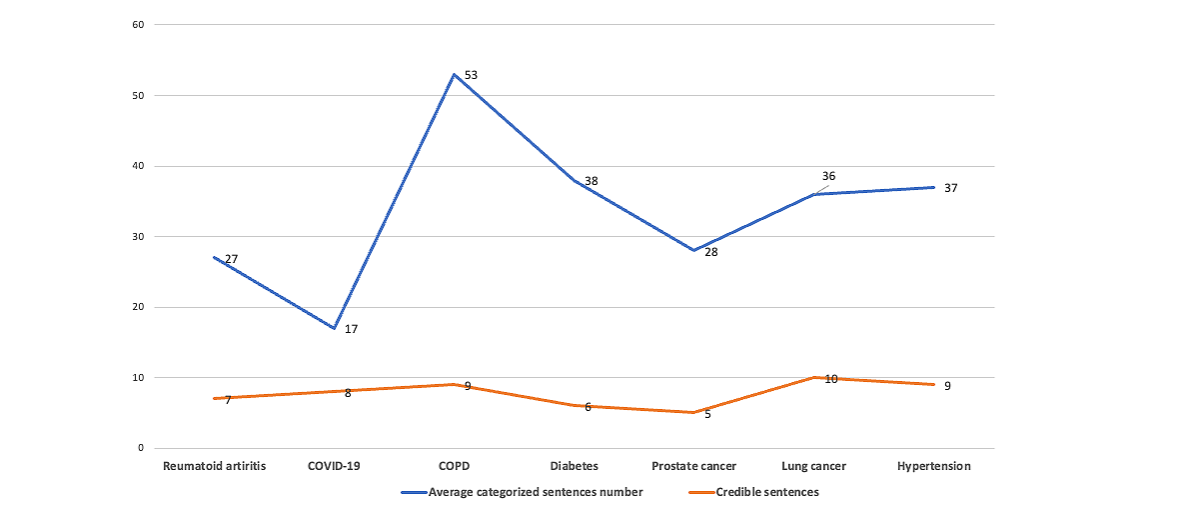
The average number of credible sentences on web pages (red line) versus the average number of all sentences on each web page (blue line). COPD: chronic obstructive pulmonary disease.

Figure 6 illustrates the average percentage of credible information found in the 5 randomly selected web pages categorized by related diseases. Credible information is defined as sentences in the web pages that were successfully matched with corresponding sentences in PubMed articles.

On average, 23% of the sentences on each web page were identified as similar to statements in the scientific literature. While this demonstrates the potential of the system to detect credible content, a significant challenge arose with false positives. Some sentences achieved a similarity score exceeding 80% but were semantically dissimilar upon closer inspection.

For instance, the following sentences from an extracted paper and a web page had a similarity score of 88% yet conveyed different meanings:

1. “Previous studies have documented residual symptoms that continue 12 weeks after the onset of acute COVID-19, known as post-acute or long COVID-19.”

2. “The acute phase of COVID itself can last for up to 14 days.”

This highlights the need for more sophisticated approaches to accurately distinguish between syntactic similarity and genuine semantic alignment.

As an illustrative example, for the rheumatoid arthritis category, we randomly selected 5 web pages, each containing an average of 27 sentences distributed across 3 thematic categories: epidemiology, semiology, and management (represented by the blue line). Among these, an average of 7 sentences per web page were deemed credible and successfully matched to corresponding statements in the scientific literature (depicted by the red line).

## Discussion

### Principal Findings

In the present pilot study, our objective was to automate aspects of the fact-checking process for online health information. While previous research [21,26] has explored automation in various stages of fact-checking, such as evidence retrieval or claim identification, this pilot serves as an initial step toward achieving full automation in the fact-checking process. Our approach includes the automation of identifying verifiable sentences through a classification process. Notably, our study used a fine-tuned BERT model, which exhibited notable efficacy in categorizing health-related sentences. Although BioBERT and SciBERT models have been reported to outperform BERT in various downstream tasks [36,37], in our investigation, the BERT model demonstrated superior performance. This discrepancy could be attributed to BERT training on general-purpose texts, such as Wikipedia or Book Corpus [35], which align more closely with the content of websites targeted at general populations. In contrast, BioBERT and SciBERT are trained on more specialized texts, such as scientific publications [36,37].

Previous research [14,31,44] has shown that the identification of claim-worthy sentences or the recognition of key information needing verification from reliable sources is a fundamental first step in automating the fact-checking process akin to our approach. This process is commonly structured as a text classification task. The previous studies used human annotators [44] or crowdsourcing [31] to tag claim-worthy sentences and trained machine learning models to classify them. A previous study [14] focused on detecting claims within news and public information, assigning each sentence a likelihood score for containing significant factual claims. Also, automating the fact-checking process is far from straightforward, as it necessitates the utilization of artificial intelligence tools to struggle with the complexity of text and context [10]. Studies often considered the problem as a binary classification to split the contents into credible or non-credible information, however, the decision is more complex since there may be several ambiguities in the sentences. In addition, several parts of the process depend on human judgment, which needs further research in the area. Building on this groundwork, our study applied a BERT-based classification approach to detect health information requiring verification and automatically proposing a sentence for this process. Previous studies relied on reviewer selections to develop claim and evidence datasets, lacking attempts to automate claim identification with real-world resources [17,18,45].

In addition, rather than constructing a manual reference dataset as the evidence for verifiable sentences, we leveraged the PubMed database as our source of truth. We automated the detection of evidence for claims made on web pages in an unsupervised approach, streamlining the verification process. This aligns with previous studies [21,26] that used PubMed publications as evidence, using transformer models to generate queries and retrieve documents from PubMed. We demonstrated the effectiveness of using transformer models to extract MeSH terms and key phrases from web page content, enabling the efficient generation of PubMed queries. This approach facilitated the retrieval of related articles from scientific references without requiring supervision. According to a previous study [14], to verify the veracity of the claims, it is crucial to translate them into queries against the reference databases. However, other studies [6,20,22] created a knowledge database as the references to compare with the claims. Notably, Sarrouti et al [6] introduced a dataset comprising evidence-claim pairs, manually annotated as SUPPORT, REFUTE, and NEUTRAL. They used BERT-based models to create a realistic testing ground for evidence-based fact-checking systems.

To assess the alignment between claim sentences and extracted references, we measured their similarity, a practice supported by [46]. This study underscores the necessity for a model in claim verification to measure the semantic similarity between claims and verified factual knowledge or references. To compare the semantic similarity, we used a transformer-based representation that converted the textual content into vectorial representation, allowing us to capture the contextual nuances of each sentence consistent with previous approaches [19,43,47]. This approach is more efficient and produces semantically richer sentence representations than simply averaging the vectors of words that appear in each sentence, and facilitates the similarity detection for the algorithms [48]. We successfully identified factual evidence for 23% of the health-related information extracted from web pages, indicating the complexity inherent in health information. Further research is required to enhance contextual comparison between claims and verified references. Also, the cosine similarity outperformed the Jaccard distance measure for comparing the claims and evidence in this study, which is different from the previous study [4], as they reported that the Jaccard distance was better at the similarity selection measure. The reason may be due to differences in the nature of the datasets in the 2 studies.

### Limitations

This study had several limitations. First, we faced a challenge in identifying sentences within the papers that closely matched the content of the web pages. Numerous methods have been devised to tackle this issue [19,43,46]; however, a comprehensive consideration of the complete meaning of sentences requires further investigation. In addition, 77% of the sentences did not have matching counterparts in the academic literature that we retrieved. Regarding this proportion, 2 possible assumptions can be made: either the sentences themselves were not valid or the algorithm was unable to locate their related counterparts. Another potential reason could be that the sentences, though addressing a common subject such as the same medical condition, exhibited variations in meaning or contextual interpretation. Consequently, it would be premature to assert that these unmatched sentences are inherently not credible, given the vast volume of published papers that renders comprehensive verification computationally infeasible. Expanding the number of selected papers for comparison could therefore increase the likelihood of identifying additional relevant sentences in the literature. Nonetheless, quantifying the proportion of credible sentences offers valuable insights to aid users in their trust assessment.

It is worth acknowledging that authors in the realm of health-related data often simplify and rephrase content to cater to their target audience, making it more challenging to identify credible references for their statements. Therefore, the researchers propose exploring other models such as text generation models as potential solutions to address this particular challenge including WordNet or sequence-to-sequence (Seq2Seq) models.

A second limitation was the sample size of the academic papers used in the comparison. Due to the extensive volume of health-related publications, the assessment was limited to a selection of 20 papers. Expanding this scope to include more papers per content type could enhance the discovery of factual evidence in PubMed publications. Thus, further investigation into paper retrieval approaches is recommended.

A third limitation was that, although the thematic categorization of web page content, such as epidemiology, semiology, and management, ensured that the generated PubMed queries were more precise and contextually relevant, the need for quality assessment of the extracted PubMed articles remains evident. While our method provides users with essential information to assess the accuracy of health information, the ultimate determination of its truthfulness may depend on individual judgment, expert evaluation, source credibility, scientific article quality (eg, journal quality, impact factor for the domain) and the contemporaneity of the information (eg, date of publication, retracted).

The retrieved articles may vary in quality, ranging from high-impact studies to potentially outdated or retracted articles that could influence the reliability of the fact-checking process and the conclusions drawn from matched content. Addressing these characteristics within an automated process remains a key challenge. In our previous research, the credibility of the sources was automatically assessed [29]. In this study, while we evaluate comparability with scientific articles, developing a credibility scoring strategy for these articles is also necessary. Combining an algorithm that evaluates website credibility and assigns a credibility score to scientific articles with 1 that determines truthfulness could significantly enhance the effectiveness of fact-checking. These models can change the structure of sentences and may improve the possibility of finding more similar sentences. Finally, while the process could not be automated entirely since each step needed human supervision for the results, the suggested techniques have the potential to substantially alleviate the human effort required to locate valid information.

### Conclusions

Our approach aimed to empower users in the decision-making process regarding the truthfulness of information by providing relevant evidence and enabling informed judgments. As a pilot, this research serves as an initial step toward exploring the feasibility of automating fact-checking processes in health information. Specifically, the methods presented here could be applied to create tailored fact-checking workflows for specific disease areas, such as diabetes, arthritis, or cancer, which were among the categories included in this study. For instance, thematic categorization (eg, management and epidemiology) could improve the precision and relevance of fact-checking tools in health care contexts. Using state-of-the-art models such as transformers may improve the performance of the model since the BERT embedding captures the meaning of the sentences [49]. The investigation also revealed that incorporating PubMed publications as a trustworthy resource can enhance the discovery of similar credible information as evidence. Finally, while the process could not be entirely automated and required human supervision, the suggested techniques demonstrate significant potential for integration into fact-checking tools. This integration could reduce the effort required to validate health information, ultimately increasing accessibility and reliability for end-users. Future work should focus on expanding the dataset and testing the approach in real-world scenarios to further refine its applicability across various health domains.

## Data Availability

The datasets generated and analyzed during this study are available from the corresponding author on reasonable request.

## Abbreviations

BERT: bidirectional encoder representations from transformers
MeSH: medical subject heading
RF: random forest
SVM: support vector machines
